# New-onset lupus nephritis and neuropsychiatric lupus following COVID-19 vaccination: A case report 

**DOI:** 10.5414/CNCS112003

**Published:** 2026-09-10

**Authors:** Paulo Simão, Micael Pompermayer, Joana Coutinho, Ernesto Rocha

**Affiliations:** 1 Department of Internal Medicine, Hospital Pêro da Covilhã, Unidade Local de Saúde da Cova da Beira, Covilhã, and; 2 Department of Nephrology, Unidade Local de Saúde de Castelo Branco, Castelo Branco, Portugal

**Keywords:** systemic lupus erythematosus, lupus nephritis, neuropsychiatric lupus, COVID-19 mRNA vaccine, autoimmune adverse events

## Abstract

Although COVID-19 mRNA vaccines have demonstrated excellent safety profiles, rare autoimmune complications – including new-onset systemic lupus erythematosus (SLE) and lupus nephritis (LN) – have been reported. We describe a case of new-onset class IV LN with neuropsychiatric lupus (NPSLE) occurring shortly after COVID-19 vaccination in a previously healthy adult. This case highlights a rare presentation of new-onset SLE manifesting with both severe renal and neuropsychiatric involvement following COVID-19 mRNA vaccination. While causality cannot be established, the temporal association and absence of alternative triggers suggest vaccine-induced immune dysregulation as a potential precipitating factor. Awareness of such atypical presentations may facilitate earlier diagnosis and management, although these events remain exceedingly uncommon relative to the overwhelming benefits of vaccination.

## Introduction 

In the aftermath of the COVID-19 pandemic, several reports have described potential side effects of mRNA vaccination, ranging from mild local reactions to more serious conditions such as myopericarditis and Guillain-Barré syndrome. Among the rarest reported adverse events are cases of ANCA-associated glomerulonephritis and lupus nephritis [1, 2]. mRNA vaccines (such as Pfizer-BioNTech and Moderna) work by instructing host cells to produce a viral protein that elicits an immune response without the use of a live virus. It is hypothesized that in genetically predisposed individuals, this immune activation may trigger aberrant responses, leading to immune-mediated diseases. 

## Case report 

A previously healthy 64-year-old man began experiencing shortness of breath and orthopnea in early October 2024, ~ 1 week after receiving his 3rd dose of the Pfizer COVID-19 vaccine. Prior to symptom onset, he led an active lifestyle, engaged in regular cycling, and had no significant past medical history. He did not smoke or use illicit drugs and consumed alcohol only occasionally. Initial evaluation by his primary care physician included referral for chest imaging and laboratory work, which was never completed. Due to progressive lower limb edema and worsening symptoms, he presented to the emergency department (ED). 

At the ED, he was deemed hemodynamically stable and treated empirically with furosemide for suspected heart failure. Later, he was sent for Internal Medicine consultation. Outpatient echocardiography, performed on January 20, revealed preserved biventricular function without valvular abnormalities or signs of pulmonary hypertension. Despite this, his symptoms progressed, prompting re-evaluation and subsequent admission for further investigation. Laboratory tests revealed normocytic anemia (Hb 9.7 g/dL), non-oliguric acute kidney injury (urea 108 mg/dL, creatinine 2.6 mg/dL), elevated LDH (407 U/L), and a significant pleural effusion on chest X-ray. C-reactive protein (CRP) was elevated at 111 mg/L. During hospitalization, he developed nephrotic-range proteinuria (6.6 g/day), hypoalbuminemia, hypertriglyceridemia, and acute confusion. He was transferred to our nephrology center in early February. 

At the time of transfer, the patient was stuporous, physically debilitated, and partially dependent for daily activities. He had been treated with quetiapine, chlorpromazine, and alprazolam for agitation. Upon initiating hemodialysis, his mental status improved, though significant limb rigidity and distal hypoesthesia persisted. These were initially attributed to medication side effects and uremia. 

### Investigations 

In addition to initial labs suggestive of nephrotic syndrome, serological tests for HIV, hepatitis B and C, and CMV were negative. Additional testing, including *Treponema pallidum* serology, was also non-reactive. Renal ultrasound showed changes consistent with acute kidney injury and an incidental cyst on the lower pole of the left kidney. Endoscopic evaluations and tumor markers (AFP, CA-19.9, CEA, CA-125) were unremarkable. Complement levels (C3, C4) were normal, while serum protein electrophoresis revealed an IgM-κ monoclonal spike. 

The patient had normocytic, normochromic anemia (Hb 8.6 g/dL), elevated CRP (111 mg/L), and procalcitonin (1.09 ng/mL). *Klebsiella pneumoniae* was isolated from urine and treated with cefuroxime. Kidney function deteriorated (urea 184 mg/dL, creatinine 5.45 mg/dL), prompting initiation of hemodialysis with ultrafiltration (Table 1). 

Autoimmune workup revealed strongly positive ANA (> 1 : 1,280), anti-dsDNA (1,295 IU/mL), anti-nDNA, and anti-SSA antibodies. Tests for anti-GBM, ANCA, anti-PLA2R, and antiphospholipid syndrome were negative. Echocardiography again showed preserved systolic function (EF 55%), with mild concentric left ventricular hypertrophy and minor valvular regurgitation. 

## Results 

Hemodiafiltration was initiated on February 6. A renal biopsy was performed the following day, and methylprednisolone pulses (1 g daily for 3 days) were started, followed by oral prednisolone (60 mg/day – approximately 1 mg/kg/day). Given the clinical picture and serologic findings, a presumptive diagnosis of systemic lupus erythematosus (SLE) with renal and neuropsychiatric involvement was made. The patient showed rapid clinical improvement and recovery of renal function, allowing discontinuation of dialysis after 6 sessions. 

Renal biopsy confirmed class IV lupus nephritis (LN) (Figure 1). Electromyography revealed axonal sensory polyneuropathy. Brain MRI showed bilateral periventricular and subcortical white matter hyperintensities, interpreted as nonspecific but consistent with neuropsychiatric lupus in this context. 

Treatment continued with glucocorticoid tapering, hydroxychloroquine (400 mg/day), and intravenous cyclophosphamide according to the Euro-Lupus regimen (500 mg every 15 days for 3 months) [3]. Enalapril was added for its antiproteinuric effect. By discharge, creatinine had improved to 1.71 mg/dL, proteinuria was reduced (5 g/L), and serum albumin increased to 2.8 g/dL. He continued to improve under outpatient follow-up with full recovery of renal function and resolution of symptoms. The patient has now a fully active, autonomous, life and exercises with regular walks. He kept some tremors of the lower limbs for ~ 3 months that subsided progressively. The polyneuropathy was solved after induction with the Euro-Lupus regimen. Serological markers showed progressive normalization following immunosuppressive therapy, with recovery of complement levels and negativization of anti-dsDNA antibodies by May 2025, remaining stable at follow-up (Table 2). 

## Discussion 

This case highlights a rare but clinically significant occurrence of class IV LN and neuropsychiatric lupus following COVID-19 mRNA vaccination in a previously healthy patient. While mRNA vaccines have demonstrated excellent safety and efficacy profiles, rare autoimmune complications have been reported, including ANCA-associated vasculitis, new-onset SLE, and LN [1, 2, 4]. Our case contributes to this growing body of literature, underscoring the need for awareness of atypical autoimmune presentations following vaccination. 

The patient’s presentation with acute kidney injury and marked nephrotic-range proteinuria, in the absence of pre-existing disease, raised suspicion for acute glomerulonephritis [5]. Acute glomerulonephritis is characterized by a sudden onset of renal impairment, often associated with hematuria, variable range proteinuria, hypertension, and edema. In this case, the nephrotic features – massive proteinuria, hypoalbuminemia, and edema – were especially prominent and clinically striking. 

Differentiating LN from primary nephrotic syndromes was crucial. Primary nephrotic syndromes generally present without systemic involvement, whereas our patient exhibited extra-renal features – most notably, neuropsychiatric symptoms suggestive of neuropsychiatric SLE (NPSLE). NPSLE remains a complex and heterogeneous manifestation of SLE. This patient’s neuropsychiatric symptoms – including stupor, confusion, and polyneuropathy – alongside MRI findings strongly supported the diagnosis despite the lack of pathognomonic biomarkers [4]. 

Although causality cannot be established, the temporal association with vaccination, absence of prior autoimmune history, and exclusion of alternative triggers support the hypothesis of immune activation as a precipitating factor. Reported cases in the literature show a similar association between mRNA vaccination and the onset of symptoms. Published reports describe de novo SLE/LN after SARS-CoV2-2 vaccination across platforms (mRNA and adenoviral vector), most often as case reports/series, with symptom onset typically within days to a few weeks after vaccination. Reported kidney histology spans proliferative (class III/IV) and membranous (class V) patterns, and presentations range from hematuria/proteinuria to nephrotic syndrome and acute kidney injury. Kim et al. [2] reported a case of class III LN in a 60-year-old woman without relevant autoimmune history, following the second dose of AstraZeneca’s COVID-19 vaccine. This case followed a similar temporal pattern to our present report. Another article, by Lemoine et al. [9], reported a case of a 68-year-old woman without a history of autoimmune conditions who developed symptoms merely 2 days after receiving the Pfizer COVID-19 vaccine, meeting criteria for SLE. Reports after AZD1222 and other vaccines similarly document new-onset SLE presenting as nephrotic syndrome/LN shortly after vaccination. From our research, LN was classified between classes III to V, without any clear predominance. This suggests an unpredictable individual response to the immunological triggers presented by these vaccines. 

Compared with most published cases, our patient is notable for the combination of severe proliferative LN (class IV) and concomitant neuropsychiatric involvement (acute confusional state/polyneuropathy with supportive MRI/EMG context), occurring shortly after a 3rd dose and requiring temporary dialysis. Neuropsychiatric de novo lupus following infection/vaccination has been described (e.g., acute psychosis/lupus cerebritis), but reports combining new-onset proliferative LN plus NPSLE remain uncommon, underscoring the atypical nature of this presentation. 

Proposed mechanisms of autoimmune activation include molecular mimicry, bystander activation, and epitope spreading [6]. Nevertheless, it is critical to emphasize that such reactions are exceedingly rare and that the benefits of vaccination far outweigh these risks. In fact, a meta-analysis by Wang et al. [7] incorporating 17 studies and over 45 million individuals revealed no significant association between vaccinations and an increased risk of SLE. Notwithstanding, a significant Korean report indicated that the risk of developing SLE post-mRNA vaccination was 1.16-fold higher than the control group, although there was no statistical significance for other autoimmune connective tissue diseases [8]. 

Management of severe SLE with multiorgan involvement requires an individualized, multidisciplinary approach. Our patient responded well to corticosteroids and immunosuppressive therapy (Euro-Lupus protocol), highlighting the importance of early diagnosis and intervention [3]. In fact, 3 months after induction following Euro-Lupus protocol with cyclophosphamide and corticosteroids, complement levels normalized and we achieved negative anti-dsDNA. For proliferative LN (class III/IV), international guidance emphasizes that induction should be followed by maintenance immunosuppression to reduce renal flares and preserve long-term kidney function. KDIGO 2024 [12] states that the total duration of initial plus maintenance immunosuppression for proliferative LN should be ≥ 36 months and recommends mycophenolic acid analogs (MPAAs), including mycophenolate mofetil (MMF), as the preferred maintenance therapy following initial treatment. EULAR also recommends that, after achieving a renal response, LN treatment should generally continue for at least 3 years, with azathioprine or MMF used as maintenance therapy following cyclophosphamide-based induction regimens. 

Randomized evidence supports this approach: in the ALMS maintenance trial [11], MMF was superior to azathioprine in maintaining renal response and preventing relapse. Other data suggest closer performance between MMF and azathioprine in some settings, but overall reinforce that ongoing maintenance therapy is standard of care. Moreover, the WIN-Lupus randomized trial [13], evaluating discontinuation after 2 – 3 years found discontinuation was not non-inferior to continuation, supporting cautious, individualized weaning strategies (often after prolonged clinical stability and sometimes guided by repeat biopsy in selected centers). In this case, given the concomitant NPSLE, continued steroid-sparing immunosuppression (e.g., MMF or azathioprine after completion of cyclophosphamide induction) was considered to reduce the risk of systemic and neurologic relapse, aligned with available recommendations and expert reviews. 

## Authors’ contributions 

Paulo Simão and Micael Pompermayer contributed equally to this work and share first authorship, including clinical data collection, literature review, and drafting of the manuscript. Joana Coutinho and Ernesto Rocha contributed to the critical revision of the manuscript for important intellectual content and supervised the work. All authors reviewed and approved the final version of the manuscript.

## Funding 

The authors received no financial support or funding for the research, authorship, and/or publication of this article. 

## Conflict of interest 

The authors declare no conflict of interest. 

**Table 1.** Laboratory evolution of the patient.

**Parameter**	**February 6**	**February 12 (end of RRT)**	**February 24** **(outpatient)**
Leucocytes (103/µL)	8.02	8.99	12.62
Hemoglobin (g/dL)	8.6	8.9	8.4
C-reactive protein (mg/L)	14.9	26.7	4.0
Urea (mg/dL)	184.0	134.0	75.0
Creatinine (mg/dL)	5.45	2.8	1.20
Sodium (mmol/L)	137.0	136.0	138.0
Total protein (g/dL)	4.3	4.0	3.6
Albumin (g/dL)	1.7	2.1	2.1
Procalcitonin (ng/mL)	1.09	0.22	0.05

RRT = renal replacement therapy.

**Figure 1.**
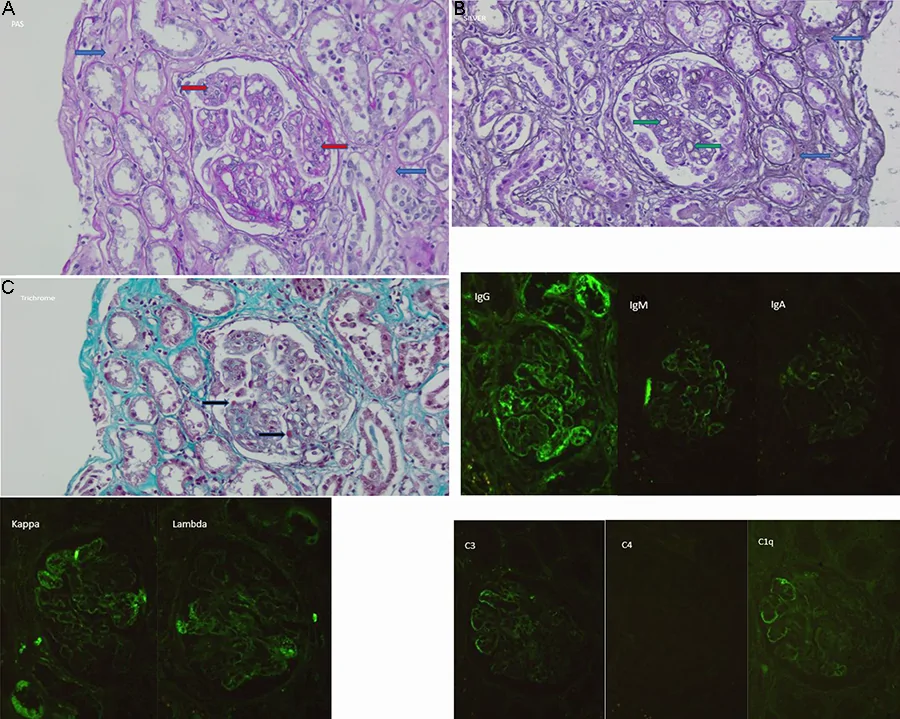
Kidney biopsy fragment on optical microscopy (×200 magnification) using periodic acid–Schiff (A), silver (B), and trichrome (C) stains and immunofluorescence (×200 magnification). Glomeruli exhibit a membranoproliferative pattern of injury, characterized by mesangial expansion and hypercellularity (red arrows), along with thickening of the glomerular basement membrane (green arrows). Subendothelial deposits and pseudo-thrombi are more distinctly visualized on trichrome staining (black arrows). There is diffuse interstitial fibroedema involving the entire cortical compartment (blue arrows), accompanied by a mild mononuclear inflammatory infiltrate, as well as acute tubular necrosis throughout the cortical compartment. Immunofluorescence shows mesangial and capillary wall deposition of IgG++; IgA+; IgM+; C3+; C1q+, κ++, and λ++.

**Table 2.** Evolution of serological markers before and after treatment.

**Date**	**Clinical phase**	**C3 (mg/dL)**	**C4 (mg/dL)**	**Anti-dsDNA (IU/mL)**	**Interpretation**
February 4, 2025	Pre treatment	72	16	179	Hypocomplementemia with high anti-dsDNA (active disease)
April 1, 2025	Early treatment	97	19	Not evaluated	Improving complement levels
May 15, 2025	Post-induction	132	36	0 (negative)	Normalized complement, negative anti-DsDNA
November 11, 2025	Follow-up	110	17	1.3	Stable/improved serology, low dsDNA
February 10, 2026	Follow-up	101	17	1.2	Stable/improved serology, low dsDNA
